# Soluble Urokinase Plasminogen Activator Receptor as a Predictor of All-Cause Death in Patients Undergoing Coronary Angiography at 10-Year Follow-Up

**DOI:** 10.3390/jcm13206158

**Published:** 2024-10-16

**Authors:** Adam Kern, Tomasz Stompór, Krystian Bojko, Ewa Sienkiewicz, Sebastian Pawlak, Krystyna Pawlak, Dariusz Pawlak, Grzegorz Poskrobko, Ewa Andrasz, Leszek Gromadziński, Rakesh Jalali, Dariusz Onichimowski, Grażyna Piwko, Artur Zalewski, Jacek Bil

**Affiliations:** 1 Department of Cardiology and Internal Medicine, School of Medicine, Collegium Medicum, University of Warmia and Mazury in Olsztyn, 10-727 Olsztyn, Poland; krystian.bojko@uwm.edu.pl (K.B.); sebastian.pawlak@uwm.edu.pl (S.P.); leszek.gromadzinski@uwm.edu.pl (L.G.); 2 Department of Cardiology, Regional Specialist Hospital in Olsztyn, 10-045 Olsztyn, Poland; stewa@mp.pl (E.S.); gposkrobko@gmail.com (G.P.); ewa8801@wp.pl (E.A.); 3 Department of Nephrology, Hypertension and Internal Medicine, School of Medicine, Collegium Medicum, University of Warmia and Mazury in Olsztyn, 10-727 Olsztyn, Poland; stompin@mp.pl; 4 Department of Monitored Pharmacotherapy, Medical University of Bialystok, 15-089 Bialystok, Poland; krystyna.pawlak@umb.edu.pl; 5 Department of Pharmacodynamics, Medical University of Bialystok, 15-089 Bialystok, Poland; dariusz.pawlak@umb.edu.pl; 6 Department of Emergency Medicine, School of Medicine, Collegium Medicum, University of Warmia and Mazury in Olsztyn, 10-727 Olsztyn, Poland; rakesh.jalali@uwm.edu.pl (R.J.); dariusz.onichimowski@uwm.edu.pl (D.O.); 7 Clinical Emergency Department, Regional Specialist Hospital in Olsztyn, 10-045 Olsztyn, Poland; 8 Clinical Department of Anaesthesiology and Intensive Care, Regional Specialist Hospital in Olsztyn, 10-045 Olsztyn, Poland; 9 Branch in Ełk, University of Warmia and Mazury in Olsztyn, 10-727 Olsztyn, Poland; grazyna.piwko@uwm.edu.pl; 10 Scanmed Cardiology Center in Ełk, 19-300 Ełk, Poland; artur.zalewski@scanmed.pl; 11 National Medical Institute of the Ministry of Interior and Administration, 02-507 Warsaw, Poland; jacekbil@mp.pl

**Keywords:** atherosclerosis, suPAR, invasive angiography, renal stenosis

## Abstract

**Background:** We aimed to explore the predictive role of soluble urokinase plasminogen activator receptor (suPAR) in patients undergoing coronary angiography by systematically evaluating its association with adverse cardiovascular events at 10 years follow-up. **Methods:** The KORONEF study was a single-center, observational, prospective study with 492 subjects included. In the multivariable Cox regression model, we checked the impact of suPAR, neutrophil elastase, myeloperoxidase, and DNase 1 on long-term outcomes. **Results:** The mean study population age was 64.4 ± 9.9 years, and there were 37.2% women. We divided the population into tertiles of suPAR levels (T1 0.793–2.135 ng/mL; T2 2.136–2.868 ng/mL; and T3 2.872–8.677 ng/mL). Patients with higher suPAR concentrations were more often females (tertile 1 vs. tertile 3: 27.4% vs. 50.6%, *p* < 0.001) and older age (60.8 ± 8.7 years vs. 68.8 ± 9.5 years, *p* < 0.001). They also characterized higher incidence of diabetes (17.7% vs. 38.0%, *p* < 0.001), previous myocardial infarction (22% vs. 44.8%, *p* < 0.001), and chronic kidney disease (3% vs. 18.4%, *p* < 0.001), but lower incidence of dyslipidemia (54.3% vs. 35.6%). The 10-year all-cause death rates were 14.6% vs. 34.1%, HR 2.68, 95% CI 1.66–4.33, *p* < 0.001 for tertile 2, and 14.6% vs. 39.9%, HR 3.24, 95% CI 2.03–5.17, *p* < 0.001 for tertile 3. The optimal cut-off suPAR value of 2.39 ng/mL provided a sensitivity of 66.9% and a specificity of 54.6% in predicting all-cause death. **Conclusions:** The association of elevated suPAR with increased mortality risk suggests its potential relevance in predicting long-term outcomes and may help inform more individualized management strategies for high-risk patients.

## 1. Introduction

The soluble urokinase plasminogen activator receptor (suPAR) is a prominent biomarker implicated in various physiological and pathological processes, including inflammation, immune response, and tissue remodeling [1]. suPAR is a cleaved form of the urokinase plasminogen activator receptor (uPAR), which is expressed on the surface of numerous cell types, such as leukocytes and endothelial cells [2]. Its elevated levels in the bloodstream have been associated with a range of diseases, particularly those characterized by increased inflammatory and thrombotic activity [3]. Given its role in modulating the fibrinolytic system and influencing cellular interactions, suPAR has emerged as a potential prognostic marker in cardiovascular disease [4,5].

In the context of coronary artery disease (CAD), predicting adverse outcomes and guiding clinical decision-making is crucial [6]. Coronary angiography remains a cornerstone in diagnosing and evaluating CAD; however, identifying patients at higher risk for complications or disease progression can enhance the management strategies [7]. Recent studies have suggested that suPAR levels might serve as a valuable biomarker for risk stratification in patients undergoing coronary angiography. Elevated suPAR concentrations have been linked to poor clinical outcomes, including myocardial infarction, heart failure, and mortality, underscoring its potential utility in this setting [8,9,10,11].

Neutrophil elastase and myeloperoxidase, both enzymes released by activated neutrophils, play crucial roles in tissue remodeling and oxidative stress, respectively. Neutrophil elastase can degrade extracellular matrix components, potentially contributing to plaque instability, while myeloperoxidase generates reactive oxygen species that exacerbate oxidative damage to vascular tissues. DNase 1, an enzyme involved in the degradation of extracellular DNA, may mitigate the formation of neutrophil extracellular traps (NETs), which have been implicated in thrombosis and atherosclerosis. The relationship between these biomarkers could reflect a synergistic contribution to the inflammatory milieu in CAD, where suPAR indicates ongoing immune activation, neutrophil elastase and myeloperoxidase contribute to tissue damage and plaque instability, and DNase 1 modulates the pro-thrombotic effects of NETs [12].

This manuscript aimed to explore the predictive role of suPAR in patients undergoing coronary angiography by systematically evaluating its association with adverse cardiovascular events. We assessed whether elevated suPAR levels correlated with the severity of coronary artery disease and predicted post-procedural complications. By elucidating the relationship between suPAR levels and clinical outcomes, our study sought to establish a foundation for incorporating suPAR as a routine biomarker in managing CAD, potentially guiding therapeutic interventions, and improving patient prognostication. 

## 2. Materials and Methods

### 2.1. Study Design and Study Population

**Figure 1:**
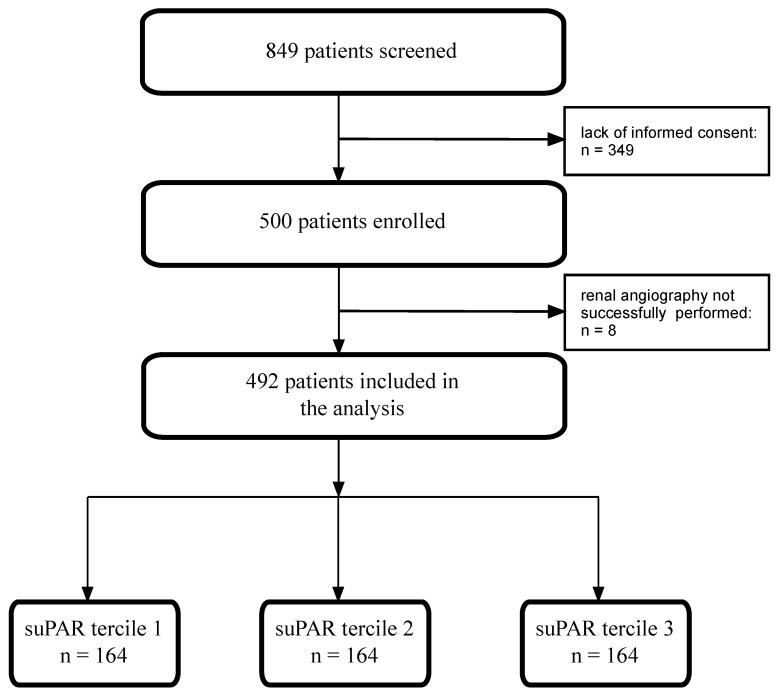
Study flowchart. suPAR—soluble urokinase plasminogen activator receptor.

As described earlier, the KORONEF study was a single-center, observational, prospective study [13]. In short, we enrolled 492 consecutive subjects who underwent both coronary angiography as well as renal angiography (Figure 1).

The study included patients hospitalized for clinical manifestations of coronary artery disease (CAD), such as chronic coronary syndrome (CCS) and acute coronary syndromes (ACS) [ST-elevation myocardial infarction (STEMI), non-ST-elevation myocardial infarction (NSTEMI), and unstable angina (UA)]. Additionally, subjects undergoing coronary angiography due to other reasons such as chronic heart failure (HF) or prior to other procedures (e.g., cardiovascular surgeries for heart valve defects, aortic aneurysm repair, pacemaker/cardioverter-defibrillator implantation) were also included. The absence of informed consent was the only exclusion criterion.

All subjects had their medical history taken, paying special attention to CAD risk factors, and underwent a physical examination. Blood samples were collected, and echocardiography was performed. Coronary angiography and simultaneous renal artery angiography were conducted on all included patients. Based on their clinical condition and the extent of CAD, patients were subsequently assigned to either conservative treatment, percutaneous coronary intervention (PCI), or coronary artery bypass grafting (CABG).

### 2.2. Data Collection

We obtained information from hospital records on diabetes, smoking status, previous myocardial infarction (MI), peripheral artery disease, dyslipidemia, prior PCI, chronic obstructive pulmonary disease (COPD), arterial hypertension, history of CABG, chronic kidney disease (CKD) with an estimated glomerular filtration rate (eGFR) of less than 60 mL/min/1.73 m^2^, and previous stroke. Additionally, we analyzed laboratory results collected at admission, which included thyroid-stimulating hormone (TSH), glucose, creatinine, uric acid, complete blood count with differential (WBC—white blood cells, RBC—red blood cells, Hgb—hemoglobin, PLT—platelets), creatine kinase (CK), CK-MB, N-terminal prohormone of brain natriuretic peptide (NT-proBNP), high-sensitivity C-reactive protein (hs-CRP), lipid profile, troponin T, and eGFR. Additionally, with commercially available enzyme-linked immunosorbent assays (ELISAs), we assessed the concentrations of suPAR, neutrophil elastase, myeloperoxidase, and DNase 1.

suPAR levels were measured using the suPARnostic^®^ AUTO Flex ELISA (ViroGates A/S, Birkerød, Denmark), an enzyme immunoassay based on a simplified double monoclonal antibody sandwich technique. In this method, samples and peroxidase-conjugated anti-suPAR were mixed on a mixing plate and incubated in optically clear microwells pre-coated with anti-suPAR. The assay utilized monoclonal antibodies from mice and rats against human suPAR. Calibration of the suPAR standard was performed against an internal Golden Standard, ensuring comparability of results across different laboratories or assay batches using the suPARnostic^®^ AUTO Flex ELISA. The lowest sensitivity was 0.4 ng/mL, and the intra-assay and inter-assay coefficients of variation (CV) of suPAR were 2.7% and 3.0%, respectively.

Human myeloperoxidase (MPO) was quantified using the Quantikine ELISA Kit (R&D Systems, Inc., Minneapolis, MN, USA), which follows a quantitative sandwich enzyme immunoassay format. A monoclonal antibody specific to human MPO was pre-coated onto a microplate to capture any MPO present in the sample. After washing away unbound substances, a horseradish peroxidase-conjugated polyclonal antibody specific to MPO was added. Following another wash, a substrate solution was applied, and color development occurred in proportion to the amount of MPO initially bound. The reaction was stopped, and the color intensity was measured.

Deoxyribonuclease 1 (DNase1) was measured using the Human DNASE1 ELISA Kit (Novus Biologicals, a Bio-Techne brand, Abingdon, UK), and neutrophil elastase was quantified using the Human Neutrophil Elastase SimpleStep ELISA Kit (Abcam, Cambridge, UK), following similar procedures to those described above.

We summarized the medications prescribed at discharge, along with echocardiographic parameters, which included left ventricular ejection fraction (LVEF), left ventricular end-diastolic diameter, interventricular septal diameter, right ventricular systolic pressure, and tricuspid annular plane systolic excursion. These measurements were performed using a commercially available diagnostic ultrasound device by experienced cardiologists, following the guidelines of the European Association of Cardiovascular Imaging [14].

Follow-up data were gathered through telephone interviews, and for missing cases, information on deaths was retrieved from the Central Statistical Office (GUS).

### 2.3. Procedure Characteristics

Patients underwent coronary angiography in conjunction with renal artery angiography. The procedure involved puncturing either the right or left femoral artery or, in select cases, the radial artery. After the puncture, a vascular sheath was inserted to allow the safe advancement of the catheters under X-ray guidance. Diagnostic angiographic catheters with a 6F diameter were used to intubate the coronary arteries, primarily with Judkins curves (JL4, JR4) and less commonly with Amplatz curves (AL1, AR1). The coronary anatomy was evaluated for the presence and location of stenoses, total occlusions, and the degree of arterial lumen narrowing.

Since femoral artery access was used for coronary angiography in over 90% of patients, adding renal artery angiography was a simple extension of the procedure. Approximately 10–20 mL of contrast medium was injected into both the right and left renal arteries, producing an angiographic image to visualize any stenoses in the renal arteries. 

### 2.4. Study Endpoints

The primary endpoint of our study was the incidence of all-cause mortality over ten years. Secondary endpoints included the rates of myocardial infarction (MI), stroke, percutaneous coronary intervention (PCI), and coronary artery bypass grafting (CABG) within the same 10-year timeframe.

### 2.5. Statistical Methods

Initially, in the multivariable Cox regression model, we checked the impact of suPAR, neutrophil elastase, myeloperoxidase, and DNase 1 on long-term outcomes. Only suPAR was an independent predictor of all-cause death at ten years. 

The data collected in the study were presented using descriptive statistics. The mean, along with the standard deviation (SD), was reported for continuous variables, and for categorical variables, the number and percentage of occurrences of each category were provided. The distribution of individual variables was compared between subgroups based on the tertiles (T1 0.793–2.135 ng/mL; T2 2.136–2.868 ng/mL; and T3 2.872–8.677 ng/mL) of the suPAR parameter value, using the Kruskal–Wallis test for continuous variables and the Fisher’s or chi-square test for categorical variables. To account for multiple comparisons between the three groups, we applied Bonferroni correction, adjusting the significance threshold accordingly to reduce the risk of Type I error.

To determine the prognostic ability of the suPAR parameter as a classifier for mortality, a ROC curve analysis was used; the area under the curve (AUC) along with a 95% confidence interval (CI), the optimal cut-off point (defined as the point on the ROC curve closest to the point of 100% sensitivity and 100% specificity), and the sensitivity and specificity at the optimal cut-off point were reported.

The analysis of time to death was conducted using survival analysis methods—Kaplan–Meier estimator with a comparison of survival curves between groups using the log-rank test, as well as univariable and multivariable Cox proportional hazards models. Variable selection for the multivariable model was performed using stepwise backward regression with the elimination of the least statistically significant variables; the initial model included all statistically significant variables in the univariable analysis. The hazard ratio (HR), along with the 95% CI and *p*-value, was reported.

To determine the relationship between the suPAR variable and the progression of atherosclerosis, the Spearman rank correlation coefficient along with the *p*-value was calculated, and suPAR levels were also compared between subgroups based on the severity of atherosclerosis.

Statistical analysis was performed using the R statistical package version 3.1.2 (R Core Team, 2014). In all analyses, the significance level was set at *p* < 0.05.

## 3. Results

### 3.1. Baseline Characteristics

Between June and December 2011, a total of 492 patients were enrolled in the study. Women made up 37.2% (n = 183) of the cohort. The mean age of the entire study population was 64.4 ± 9.9 years (range: 30–89 years), and the mean body mass index (BMI) was 27.9 ± 4.3 kg/m^2^. Patients with higher suPAR concentrations were more often females (tertile 1 vs. tertile 3: 27.4% vs. 50.6%, *p* < 0.001) and older age (60.8 ± 8.7 years vs. 68.8 ± 9.5 years, *p* < 0.001). They also characterized a higher incidence of diabetes (17.7% vs. 38.0%, *p* < 0.001), previous MI (22% vs. 44.8%, *p* < 0.001), and chronic kidney disease (3% vs. 18.4%, *p* < 0.001), but a lower incidence of dyslipidemia (54.3% vs. 35.6%). The left ventricular ejection fraction was also lower in patients with higher suPAR levels (55.5 ± 11.7% vs. 50.4 ± 11.8%, *p* < 0.001) (Table 1).

Patients with higher suPAR concentrations characterized higher levels of glucose (*p* < 0.001), creatinine (*p* = 0.002), hs-CRP (*p* < 0.001), as well as neutrophile elastase (*p* < 0.001) and myeloperoxidase (*p* < 0.001), and lower levels of hemoglobin (*p* < 0.001), eGFR (*p* < 0.001), and total cholesterol (*p* = 0.022) (Table 2).

### 3.2. Periprocedural and Discharge Characteristics

The planned CAD diagnostics was the most common indication for coronary angiography (n = 290, 59.4%). Coronary angiography performed in 122 (25%) patients did not reveal any angiographically significant lesions in coronary arteries, while in 67 (13.7%) patients, the presence of three-vessel CAD was detected, including involvement of the left main stem (LM, n = 22 [4.5%]). The disease advancement increased with suPAR levels. Additionally, 208 (45.7%) patients were qualified for percutaneous revascularization. Interestingly, the no flow/slow phenomenon rate was also the highest in the highest tertile of suPAR concentration (3.2% vs. 8.8%, *p* = 0.012) (Table 3).

Patients received treatment in accordance with standards, with 90.0% of patients receiving acetylsalicylic acid at discharge, 91.1%—an angiotensin-converting enzyme (ACE) inhibitor/angiotensin receptor antagonist, 92.1%—a beta-blocker, and 93.1%—a statin. Patients with higher suPAR concentrations received more frequently clopidogrel (*p* = 0.005), Ca-blocker (*p* = 0.004), loop diuretics (*p* < 0.001), mineralocorticoid receptor antagonist (*p* = 0.015), vitamin K antagonist (*p* = 0.020), and insulin (*p* < 0.001) (Table 4).

### 3.3. Ten-Year Follow-Up Data

**Figure 2:**
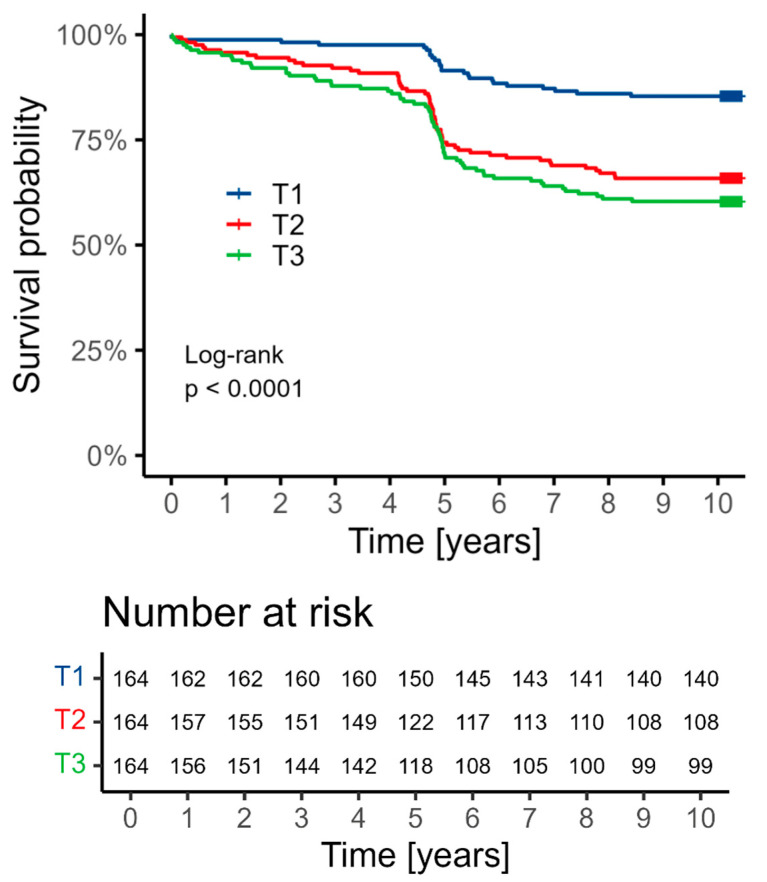
Kaplan–Meier curve showing survival depending on the tertile of suPAR concentrations. The distribution of individual variables was compared between subgroups based on the tertiles: T1 0.793–2.135 ng/mL; T2 2.136–2.868 ng/mL; and T3 2.872–8.677 ng/mL.

The median follow-up period was 10.2 years (min. 5.9 years; max. 10.3 years). In the whole population, all-cause death was observed in 29.5% of patients, MI—in 11.4%, and stroke—in 4.9%. Patients with higher suPAR concentrations characterized higher rates of all-cause death. This relationship was visible for tertile 2 as well as tertile 3 in comparison to tertile 1. The all-cause death rates were 14.6% vs. 34.1%, HR 2.68, 95% CI 1.66–4.33, *p* < 0.001 for tertile 2, and 14.6% vs. 39.9%, HR 3.24, 95% CI 2.03–5.17, *p* < 0.001 for tertile 3 (Table 5, Figure 2).

### 3.4. suPAR Threshold Identifications

**Figure 3:**
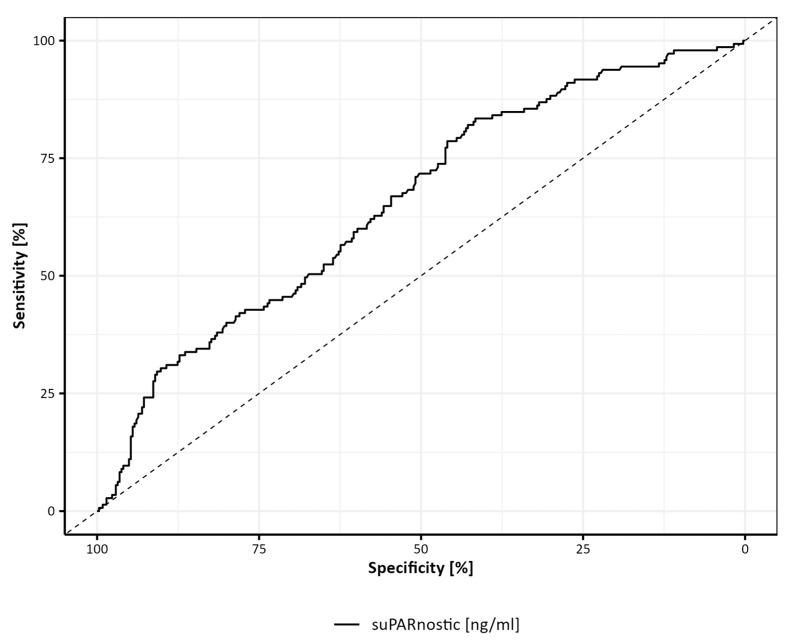
ROC curve for suPAR cut-off point.

Based on these results (almost no prognostic difference between suPAR tercile 2 and tercile 3), we aimed to identify the best cut-off point for suPAR concentration in predicting all-cause death. The ROC curve analysis for suPAR concentration in predicting 10-year all-cause mortality yielded an AUC of 65.6% (95% CI: 60.4–70.8%), indicating a moderate discriminative ability. The optimal cut-off value of 2.39 ng/mL provided a sensitivity of 66.9% and a specificity of 54.6% (Figure 3). 

### 3.5. suPAR Concentration and CAD Advancement

**Figure 4:**
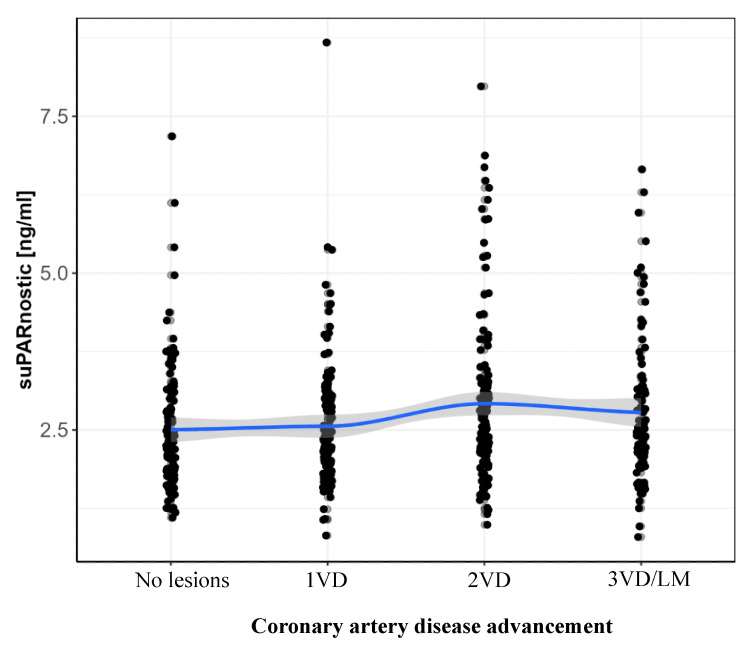
suPAR concentration values depending on the advancement of coronary artery disease. 1VD—one vessel disease, 2VD—two-vessel disease, 3VD—three-vessel disease, LM—left main.

The suPAR concentration correlated with CAD advancement. The mean concentration of suPAR in patients with no lesions was 2.50 ± 0.98 ng/mL, with 1VD—2.56 ± 0.98 ng/mL, with 2VD—2.92 ± 1.29 ng/mL, and with 3VD/LM—2.78 ± 1.18 ng/mL. The analysis showed a Spearman rank correlation coefficient of 0.117 between suPAR levels and coronary angiography findings, with a *p*-value of 0.010. This result suggested a weak but statistically significant positive correlation between suPAR concentration and the extent of changes observed in coronary angiography (Figure 4).

### 3.6. Cox Analysis

Finally, we analyzed predictive factors for all-cause death at ten years. The multivariable analysis results are depicted in Table 6 (univariable analysis is presented in Supplementary Table S1). In the multivariable model, statistically significant predictors included age 65–75 years (HR 3.58), age > 75 years (HR 9.68), diabetes (HR 1.61), previous MI (HR 1.64), CKD (HR 2.32), LV ejection fraction > 60% (HR 0.38), as well as suPAR (for tertile 2—HR 1.68, and for tertile 3—HR 3.45) (Table 6). 

## 4. Discussion

Our study demonstrated that a suPAR level greater than 2.21 ng/mL served as a significant independent predictor of mortality over a 10-year follow-up period in patients undergoing simultaneous coronary and renal angiography. This finding highlighted the potential utility of suPAR as a prognostic biomarker in this patient population. The association of elevated suPAR with increased mortality risk underscored its relevance in predicting long-term outcomes and could guide more individualized management strategies for high-risk patients.

Our study’s findings indicated a correlation between suPAR levels and the advancement of atherosclerosis, as evidenced by the relationship between suPAR and the extent of CAD. The data showed a notable variation in suPAR levels across different degrees of atherosclerosis, with a *p*-value of 0.022, suggesting that suPAR levels were indeed associated with the severity of coronary artery disease. Specifically, patients with more advanced atherosclerosis, such as those with two-vessel disease (2-VD) and three-vessel or left main (3-VD/LM) disease, tended to have higher suPAR levels compared to those with less severe or no detectable coronary lesions.

Recently, Guan et al. showed that suPAR was associated with cardiovascular calcification in peritoneal dialysis patients, particularly among the elderly. Serum suPAR levels were strongly correlated with the severity of abdominal aortic calcification, coronary artery calcification, and cardiac valvular calcification in these patients. Those with higher suPAR levels had a greater incidence of cardiovascular calcifications. The ROC curve analysis demonstrated that suPAR had a predictive value for cardiovascular calcifications (AUC = 0.651), with a particularly strong predictive value for cardiac valvular calcifications (AUC = 0.828) [15].

The observed association supports the hypothesis that suPAR is involved in the inflammatory and fibrinolytic processes underlying atherosclerosis. suPAR is known to be a marker of systemic inflammation, which plays a critical role in the development and progression of atherosclerotic lesions. Elevated suPAR levels in more advanced stages of atherosclerosis may reflect increased inflammatory activity and endothelial dysfunction, which are hallmarks of severe CAD. These findings align with existing literature highlighting inflammation’s role in atherosclerotic disease progression. There are also studies showing the impact of suPAR on endothelial dysfunction of coronary arteries but without significant epicardial atherosclerosis [16]. Al-Badri et al. showed that in patients with non-obstructive coronary arteries elevated suPAR (>2.5 ng/mL) and hsTnI (>2.7 pg/mL) levels were independent predictors of all-cause mortality (HR 3.2 [95% CI, 1.8–5.7] and 1.3 [95% CI, 1.0–1.7], respectively; both *p* < 0.04) and major adverse cardiovascular events (HR of 2.7 [95% CI, 1.4–5.4] and 1.5 [95% CI, 1.2–2.0], respectively; both *p* < 0.002). Biomarker risk scores of 1 and 2 were associated with 19-fold and 14-fold increases in the risk of death and major adverse cardiovascular events, respectively, compared to a biomarker risk score of 0 [17]. Bergamaschi et al. also showed that increased inflammatory response observed in cardiac magnetic resonance is also associated with a worse prognosis. Elevated T2 values in the non-infarcted myocardium following STEMI were independently linked to poorer cardiovascular outcomes, primarily due to an increased risk of myocardial infarction [18].

We showed in the ROC analysis that suPAR levels offered a moderate predictive capability for mortality, with an area under the curve (AUC) of 65.6% (95% CI: 60.4–70.8%). This indicated that suPAR is a useful, though not perfect, biomarker for assessing mortality risk in this patient cohort. The optimal cut-off value of 2.39 ng/mL shows a balance between sensitivity (66.9%) and specificity (54.6%), suggesting that while suPAR can effectively identify patients at increased risk, there is room for improving its predictive accuracy. The ROC analysis reinforces the clinical utility of suPAR as a prognostic tool. The sensitivity of 66.9% indicates that suPAR is relatively effective at identifying individuals who will experience adverse outcomes, which is crucial for early intervention. However, the specificity of 54.6% suggests that there is a substantial rate of false positives, meaning that some patients identified as high risk may not experience mortality within the follow-up period.

Incorporating suPAR into clinical practice could enhance risk stratification, particularly in patients with ambiguous prognostic indicators. Given its moderate AUC, suPAR should be used in conjunction with other established risk factors and clinical variables to provide a more comprehensive assessment. Combining suPAR with other biomarkers or risk scores may improve its predictive performance and assist in tailoring more personalized treatment strategies [19].

Li et al. showed in the meta-analysis that patients with CAD who had elevated suPAR levels faced a significantly increased risk of all-cause mortality (HR = 2.24; 95% CI 1.97–2.55) and cardiovascular mortality (HR = 2.02; 95% CI 1.58–2.58). However, there was no significant increase in the risk of experiencing other major cardiovascular events (HR = 1.63; 95% CI 0.86–3.11) [20]. Furthermore, Walter et al. showed that the prognostic discrimination of suPAR was moderate for cardiovascular death (AUC = 0.72, 95% CI 0.62–0.81) and all-cause death (AUC = 0.72, 95% CI 0.65–0.79) over two years. SuPAR remained a significant predictor of all-cause mortality in multivariable Cox regression analysis, with a HR of 1.96 (*p* = 0.001) (suPAR cut-off—3.2 ng/mL) [21]. In other studies, the cut-off value was even higher, with the range of 3.5–4.5 ng/mL. 

The multivariable Cox regression analysis confirmed the robustness of suPAR as a predictor of mortality even after adjusting for various clinical and demographic factors. Notably, suPAR’s predictive value remained significant irrespective of other established risk factors, such as age, diabetes, and MI history. Nevertheless, age, in particular, showed a dose-response relationship with mortality risk, consistent with previous studies linking advanced age with worse cardiovascular outcomes. Diabetes and a history of myocardial infarction were associated with higher mortality risk, aligning with established knowledge about their adverse impact on long-term prognosis. However, suPAR’s predictive power remained strong even when these variables were accounted for, suggesting that suPAR could be a valuable tool in identifying patients at the highest risk of adverse outcomes.

Future studies should explore the mechanisms underlying the relationship between suPAR and mortality and assess how this biomarker can be integrated into existing clinical decision-making frameworks. This is even more vital since Wholwend et al. showed interesting findings regarding suPAR in young and healthy adults [22]. Females exhibited higher plasma suPAR levels than males (1.73 ng/mL vs. 1.50 ng/mL; *p* < 0.001). There was a significant inverse correlation between plasma suPAR levels and HDL cholesterol in both men (*p* = 0.001) and women (*p* < 0.001). Additionally, male and female smokers had notably higher plasma suPAR levels (*p* < 0.001). Among men, there was an inverse correlation between a healthy lifestyle score and suPAR levels (*p* = 0.001) and a positive correlation between the Framingham score and suPAR levels (*p* < 0.001), whereas no such correlations were observed in women. In female participants, cholesterol levels (*p* = 0.001) and HbA1c (*p* = 0.008) were significantly correlated with plasma suPAR levels. These findings indicate that plasma suPAR levels are strongly linked to certain cardiovascular risk factors. Moreover, these results are quite similar to our findings, but our study was carried out in a diseased population. Moreover, others showed that suPAR levels are linked to cardiac deaths and MI independently of troponin or hsCRP levels, as well as renal function [23].

Therefore, further research should focus on validating the prognostic value of suPAR across diverse patient populations and in different clinical settings. Additionally, exploring the mechanistic pathways linking suPAR to adverse outcomes could provide insights into its role in disease progression. An improved understanding of suPAR’s biological functions and its interaction with other markers might enhance its utility as a predictive tool and inform strategies to mitigate long-term mortality risks in cardiovascular and renal disease management [24].

Piccioni et al. recently proposed a multi-marker approach in patients with chest pain in the ED [25]. They suggested that tumorigenicity suppression-2 (sST2) and suPAR, along with hsTnI, were useful in predicting outcomes in cardiovascular patients with ACS, providing additional insights into endothelial damage. They identified two patient groups: a positive group (112 patients) with elevated levels of hsTnI, sST2 > 24.19 ng/mL, and suPAR > 2.9 ng/mL, all diagnosed with ACS, and a negative group (136 patients) with lower levels of hsTnI, suPAR < 2.9 ng/mL, and sST2 < 24.19 ng/mL. During the 12-month follow-up, no adverse events occurred in the negative group. Among patients in the intermediate group, those with hsTnI between 6 ng/L and the ischemic threshold, sST2 > 29.1 ng/mL, and suPAR > 2.9 ng/mL had the highest likelihood of adverse events during follow-up. In contrast, those with sST2 < 24.19 ng/mL and suPAR < 2.9 ng/mL experienced better outcomes, with no adverse events observed at 12 months. Interestingly, Pruc et al. showed that suPAR levels increased in patients with acute coronary syndrome [26], and others also built more complex multi-biomarker strategies [27].

## 5. Study Limitations

The study had several limitations. One of the limitations was the inclusion of not only patients with CAD but also all individuals undergoing coronary angiography, leading to a less homogeneous study group. Moreover, it is important to note that the exclusive use of clopidogrel, rather than newer P2Y12 receptor inhibitors, may have been associated with poorer outcomes.

## 6. Conclusions

Our study suggests that a suPAR level greater than 2.21 ng/mL is a potential independent predictor of mortality over a 10-year follow-up period in patients undergoing simultaneous coronary and renal angiography. However, given the study’s limitations and design, further validation of this cut-off in larger cohorts is required. While elevated suPAR levels appear associated with increased mortality risk, caution should be exercised in generalizing these findings, and additional research is needed to confirm its utility as a prognostic biomarker for long-term outcomes and to refine its role in guiding individualized management strategies for high-risk patients.

## Figures and Tables

**Table 1:** Baseline characteristics based on suPAR concentration.

Parameter	Total N = 492	Tertile 1 N = 164	Tertile 2 N = 164	Tertile 3 N = 164	*p*
Females	184 (37.4%)	45 (27.4%)	56 (34.1%)	83 (50.6%)	<0.001
Age [years]	64.4 ± 9.8	60.8 ± 8.7	63.6 ± 9.7	68.8 ± 9.5	<0.001
BMI [kg/m^2^]	27.9 ± 4.3	28.0 ± 4.0	27.7 ± 4.2	27.9 ± 4.7	0.845
Arterial hypertension	366 (74.8%)	117 (71.3%)	125 (77.2%)	124 (76.1%)	0.436
Dyslipidemia	225 (46.0%)	89 (54.3%)	78 (48.1%)	58 (35.6%)	0.003
Diabetes	126 (25.8%)	29 (17.7%)	35 (21.6%)	62 (38.0%)	<0.001
Obesity	147 (30.1%)	53 (32.3%)	45 (27.8%)	49 (30.1%)	0.671
Previous MI	154 (31.5%)	36 (22.0%)	45 (27.8%)	73 (44.8%)	<0.001
Previous stroke	32 (6.6%)	4 (2.4%)	15 (9.3%)	13 (8.0%)	0.029
Peripheral artery disease	24 (4.9%)	6 (3.7%)	6 (3.7%)	12 (7.4%)	0.206
Chronic kidney disease	47 (9.6%)	5 (3.0%)	12 (7.4%)	30 (18.4%)	<0.001
Previous CABG	21 (4.3%)	4 (2.4%)	5 (3.1%)	12 (7.4%)	0.058
Previous PCI	103 (21.1%)	31 (18.9%)	33 (20.4%)	39 (23.9%)	0.519
Echocardiography results					
LV ejection fraction [%]	52.5 ± 11.5	55.5 ± 11.7	51.9 ± 10.7	50.4 ± 11.8	<0.001
RVSP [mmHg]	38.9 ± 10.3	42.3 ± 11.2	38.0 ± 8.0	38.9 ± 11.4	0.785
TAPSE [mm]	17.4 ± 4.2	21.0 ± 1.1	22.0 ± 1.0	14.5 ± 2.4	0.034

Results presented as mean ± standard deviation: BMI—body mass index; MI—myocardial infarction; CABG—coronary artery bypass grafting; PCI—percutaneous coronary intervention; LV—left ventricular; RVSP—right ventricular systolic pressure; TAPSE—tricuspid annular plane systolic excursion.

**Table 2:** Biochemical tests based on suPAR concentration.

Parameter	Total N = 492	Tertile 1 N = 164	Tertile 2 N = 164	Tertile 3 N = 164	*p*
Hemoglobin [g/dL]	13.9 ± 1.4	14.2 ± 1.2	14.1 ± 1.3	13.3 ± 1.5	<0.001
Glucose [mg/dL]	116.4 ± 39.6	106.6 ± 26.2	116.0 ± 34.7	127.0 ± 51.4	<0.001
Creatinine [mg/dL]	1.0 ± 0.6	0.9 ± 0.2	0.9 ± 0.2	1.2 ± 0.9	0.002
eGFR [mL/min]	81.7 ± 24.2	87.1 ± 18.2	85.4 ± 26.0	72.4 ± 25.3	<0.001
K^+^ [mmol/L]	4.4 ± 0.5	4.3 ± 0.4	4.3 ± 0.4	4.5 ± 0.5	0.058
Na^+^ [mmol/L]	140.7 ± 2.8	141.0 ± 2.5	140.7 ± 2.8	140.4 ± 3.2	0.313
Total cholesterol [mg/dL]	183.2 ± 51.1	187.3 ± 58.1	189.2 ± 49.1	173.2 ± 43.8	0.022
LDL [mg/dL]	109.6 ± 44.5	112.0 ± 51.3	113.5 ± 41.7	103.2 ± 38.9	0.120
HDL [mg/dL]	53.5 ± 17.4	55.4 ± 17.6	54.5 ± 20.0	50.5 ± 13.9	0.058
Triglycerides [mg/dL]	139.6 ± 91.6	135.6 ± 91.6	146.5 ± 109.8	142.5 ± 109.8	0.463
TSH [μIU/mL]	2.1 ± 4.8	1.9 ± 1.8	1.7 ± 1.8	2.8 ± 8.0	0.844
hs-CRP [mg/L]	1.2 ± 4.9	0.5 ± 1.1	1.2 ± 4.2	1.8 ± 7.4	<0.001
Neutrophile elastase [ng/mL]	416.20 ± 358.88	313.54 ± 240.72	416.91 ± 346.73	518.14 ± 434.43	<0.001
Myeloperoxidase [ng/mL]	250.83 ± 278.12	178.23 ± 174.11	246.03 ± 225.47	328.23 ± 374.83	<0.001
DNASE1 [ng/mL]	1.77 ± 13.95	0.75 ± 1.18	1.77 ± 9.72	2.80 ± 22.09	0.419

Results presented as mean ± standard deviation; HDL—high-density lipoprotein; LDL—low-density lipoprotein; TSH—thyroid-stimulating hormone; hs-CRP—high sensitivity C-reactive protein.

**Table 3:** Periprocedural data based on suPAR concentration.

Parameter	Total N = 492	Tertile 1 N = 164	Tertile 2 N = 164	Tertile 3 N = 164	*p*
Coronary angiography indications					
Planned coronary artery disease diagnostics	290 (59.4%)	105 (64.4%)	94 (58.0%)	91 (55.8%)	0.181
Acute coronary syndrome	170 (34.6%)	49 (29.9%)	61 (37.2%)	60 (36.6%)	
Heart failure diagnostics	4 (0.8%)	1 (0.6%)	2 (1.2%)	1 (0.6%)	
Pacemaker/ICD qualification	4 (0.8%)	1 (0.6%)	0 (0.0%)	3 (1.8%)	
Cardiovascular surgery (heart valve defect, ascending aorta aneurysm)	24 (4.9%)	8 (4.9%)	7 (4.3%)	9 (5.5%)	
Coronary angiography results					
No significant atherosclerotic lesions *	122 (25.0%)	51 (31.3%)	37 (22.8%)	34 (20.9%)	0.042
One-vessel disease	140 (28.7%)	50 (30.7%)	47 (29.0%)	43 (26.4%)	
Two-vessel disease	137 (28.1%)	35 (21.5%)	47 (29.0%)	55 (33.7%)	
Three-vessel disease	67 (13.7%)	24 (14.7%)	21 (13.0%)	22 (14.7%)	
Left main stem	22 (4.5%)	3 (1.8%)	10 (6.2%)	9 (5.5%)	
Qualification for revascularization					
Pharmacological treatment	156 (34.3%)	63 (40.4%)	47 (30.9%)	46 (31.3%)	0.225
PCI	208 (45.7%)	62 (39.7%)	78 (51.3%)	68 (46.3%)	
CABG	91 (20.0%)	31 (19.9%)	27 (17.8%)	33 (22.4%)	
Location of lesions treated by PCI					
Left main stem	1 (0.4%)	0 (0.0%)	0 (0.0%)	1 (1.2%)	0.610
Left anterior descending artery/ diagonal branches	90 (38.3%)	30 (44.2%)	34 (39.5%)	26 (32.1%)	
Left circumflex artery/marginal branches	58 (24.7%)	15 (22.4%)	22 (25%)	21 (25.9%)	
Intermediate artery	4 (1.7%)	1 (1.5%)	1 (1.2%)	2 (2.5%)	
Right coronary artery	80 (34.0%)	22 (32.9%)	29 (32.9%)	29 (35.8%)	
Venous graft	2 (0.9%)	0 (0.0%)	0 (0.0%)	2 (2.5%)	
TIMI after PCI					
0	10 (4.2%)	3 (4.3%)	1 (1.2%)	6 (7.3%)	0.123
1	2 (0.8%)	0 (0.0%)	2 (2.4%)	0 (0.0%)	
2	1 (0.4%)	0 (0.0%)	1 (1.2%)	0 (0.0%)	
3	223 (94.5%)	66 (95.7%)	81 (95.3%)	76 (92.7%)	
Periprocedural complications (PCI)					
No reflow/slow reflow	8 (3.8%)	2 (3.2%)	0 (0.0%)	6 (8.8%)	0.012
Stent thrombosis	1 (0.5%)	1 (1.6%)	0 (0.0%)	0 (0.0%)	

* No atherosclerotic lesions or lesions <40%, TIMI—thrombolysis in myocardial infarction; PCI—percutaneous coronary intervention, CABG—coronary artery bypass grafting, ICD—implantable cardioverter-defibrillator.

**Table 4:** Medications at discharge based on suPAR concentration.

Parameter	Total N = 492	Tertile 1 N = 164	Tertile 2 N = 164	Tertile 3 N = 164	*p*
Acetylsalicylic acid	442 (90.0%)	143 (87.2%)	149 (91.4%)	150 (91.5%)	0.335
Clopidogrel	301 (61.3%)	84 (51.2%)	107 (65.6%)	110 (67.1%)	0.005
ACE inhibitor	426 (86.8%)	138 (84.1%)	144 (88.3%)	144 (87.8%)	0.475
Angiotensin antagonist	21 (4.3%)	10 (6.1%)	7 (4.3%)	4 (2.4%)	0.262
Beta-blocker	452 (92.1%)	153 (93.3%)	146 (89.6%)	153 (93.3%)	0.356
Ca-blocker	124 (25.3%)	29 (17.7%)	55 (33.7%)	40 (24.4%)	0.004
Statin	457 (93.1%)	151 (92.1%)	153 (93.9%)	153 (93.3%)	0.808
Fibrate	17 (3.5%)	7 (4.3%)	7 (4.3%)	3 (1.8%)	0.374
Loop diuretic	89 (18.1%)	9 (5.5%)	31 (19.0%)	49 (29.9%)	<0.001
Thiazide	50 (10.2%)	17 (10.4%)	14 (8.6%)	19 (11.6%)	0.666
Mineralocorticoid receptor antagonist	59 (12.0%)	10 (6.1%)	23 (14.1%)	26 (15.9%)	0.015
Vitamin K antagonist	37 (7.5%)	5 (3.0%)	14 (8.6%)	18 (11.0%)	0.020
Insulin	44 (9.0%)	7 (4.3%)	11 (6.7%)	26 (15.9%)	<0.001

ACE—angiotensin-converting enzyme.

**Table 5:** The outcomes in 10-year follow-up.

Endpoint	Total N = 492	Tertile 1 N = 164	Tertile 2 N = 164	Tertile 3 N = 164	*p*
Death	145 (29.5%)	24 (14.6%)	56 (34.1%)	65 (39.9%)	<0.001
MI	56 (11.4%)	17 (10.4%)	21 (12.8%)	18 (11.0%)	0.773
Stroke	24 (4.9%)	7 (4.3%)	7 (4.3%)	10 (6.1%)	0.665
CABG	37 (7.5%)	19 (11.6%)	10 (6.1%)	8 (4.9%)	0.051
PCI	81 (16.5%)	32 (19.5%)	26 (15.9%)	23 (14.1%)	0.405

MI—myocardial infarction; CABG—coronary artery bypass grafting; PCI—percutaneous coronary intervention.

**Table 6:** Factors predicting death in the 10-year follow-up—multivariable Cox analysis.

	Study Population N = 492 (%)		
Parameter	HR	95% CI	*p*-Value
**Age:**			
65–75 years	3.58	1.57, 8.16	0.002
75–90 years	9.68	4.27, 21.9	<0.001
**Diabetes**	1.61	1.07, 2.41	0.022
**Previous myocardial infarction**	1.64	1.09, 2.47	0.018
**Chronic kidney disease**	2.32	1.41, 3.82	<0.001
**Coronary angiography indications:**			
STEMI	1.77	1.03, 3.04	0.040
**Left ventricular ejection fraction**			
[40, 50]	0.46	0.28, 0.76	0.002
[50, 60]	0.55	0.31, 0.95	0.033
>60	0.38	0.21, 0.71	0.002
**LDL cholesterol**			
[100, 129]	0.61	0.35, 1.06	0.080
**suPAR**			
T2	1.68	0.99, 4.33	0.073
T3	3.45	2.11, 5.92	<0.001

HR = hazard ratio, CI = confidence interval, STEMI—ST-elevation myocardial infarction.

## Data Availability

Data are available from the corresponding author on request.

## Supplementary Materials

The following supporting information can be downloaded at: https://www.mdpi.com/article/10.3390/jcm13206158/s1, Supplementary Tables S1. Univariable Cox regression for death in the whole population.
