# Supplementary material for: Soluble Urokinase Plasminogen Activator Receptor as a Predictor of All-Cause Death in Patients Undergoing Coronary Angiography at 10-Year Follow-Up
Source: J Clin Med. 2024 Oct 16;13(20):6158. doi: 10.3390/jcm13206158 (PMC11508504; doi:10.3390/jcm13206158)
Supplement: Supplementary file 1 [file jcm-13-06158-s001.zip › jcm-3211571-supplementary.pdf]

Suplementary Table S1: Cox regression

	Univariable COX regression			Multivariable COX regression		
**Characteristic**	**HR[1]**	**95% CI[1]**	**p-value**	**HR[1]**	**95% CI[1]**	**p-value**
**Sex**						
Female	—	—				
Male	1.02	0.73, 1.43	0.9			
**Age**						
(30,55]	—	—		—	—	
(55,60]	1.81	0.83, 3.93	0.13	2.10	0.87, 5.07	0.10
(60,65]	1.45	0.63, 3.31	0.4	1.53	0.58, 4.04	0.4
(65,75]	3.67	1.80, 7.48	<0.001	3.58	1.57, 8.16	0.002
(75,90]	8.71	4.29, 17.7	<0.001	9.68	4.27, 21.9	<0.001
**BMI**						
<25	—	—				
[25,29.9]	0.94	0.60, 1.48	0.8			
[30,34.9]	0.76	0.43, 1.34	0.3			
≥35	0.66	0.26, 1.71	0.4			
**Diabetes**						
No	—	—		—	—	
Yes	2.01	1.43, 2.82	<0.001	1.61	1.07, 2.41	0.022
**Hypertension**						
No	—	—				
Yes	1.15	0.78, 1.70	0.5			
**Smoking**						
No	—	—				
Yes	0.67	0.45, 1.00	0.048			
**Obesity**						
No	—	—				
Yes	1.03	0.72, 1.46	0.9			

	Univariable COX regression			Multivariable COX regression		
**Characteristic**	**HR[1]**	**95% CI[1]**	**p-value**	**HR[1]**	**95% CI[1]**	**p-value**
**Dyslipidemia**						
No	—	—				
Yes	0.65	0.46, 0.91	0.011			
**Prior MI**						
No	—	—		—	—	
Yes	2.02	1.45, 2.80	<0.001	1.64	1.09, 2.47	0.018
**Stroke**						
No	—	—				
Yes	2.79	1.72, 4.52	<0.001			
**PAD**						
No	—	—				
Yes	1.72	0.93, 3.19	0.082			
**Dialysis**						
No	—	—				
Yes	0.00	0.00, Inf	>0.9			
**CKD**						
No	—	—		—	—	
Yes	2.71	1.78, 4.12	<0.001	2.32	1.41, 3.82	<0.001
**Prior CABG**						
No	—	—				
Yes	1.81	0.95, 3.45	0.070			
**Prior PCI**						
No	—	—				
Yes	1.30	0.89, 1.90	0.2			
**STEMI**						
No	—	—				
Yes	1.10	0.71, 1.70	0.7			

	Univariable COX regression			Multivariable COX regression		
**Characteristic**	**HR[1]**	**95% CI[1]**	**p-value**	**HR[1]**	**95% CI[1]**	**p-value**
**NSTEMI**						
No	—	—				
Yes	1.27	0.81, 2.01	0.3			
**UA**						
No	—	—				
Yes	1.15	0.67, 1.97	0.6			
**Cardiac arrest**						
No	—	—				
Yes	3.36	1.38, 8.21	0.008			
**AF**						
No	—	—				
Yes	2.66	1.68, 4.24	<0.001			
**Disease advancement**						
3-VD	—	—				
LM	1.25	0.57, 2.71	0.6			
**BMS stent number**						
0	—	—				
1	1.00	0.68, 1.46	>0.9			
2	1.65	0.83, 3.26	0.2			
3	1.25	0.17, 8.96	0.8			
**DES stent number**						
0	—	—				
1	0.63	0.38, 1.06	0.083			
2	0.74	0.18, 2.99	0.7			
3	0.00	0.00, Inf	>0.9			
**POBA**						

	Univariable COX regression			Multivariable COX regression		
**Characteristic**	**HR[1]**	**95% CI[1]**	**p-value**	**HR[1]**	**95% CI[1]**	**p-value**
0	—	—				
1	0.86	0.55, 1.35	0.5			
2	2.78	0.39, 19.9	0.3			
**TIMI after PCI**						
0	—	—				
1	0.74	0.09, 6.34	0.8			
2	0.00	0.00, Inf	>0.9			
3	0.43	0.17, 1.08	0.072			
**Indication to coronary angiography**						
CAD	—	—		—	—	
NSTEMI	1.10	0.68, 1.77	0.7	0.99	0.59, 1.69	>0.9
STEMI	1.21	0.77, 1.92	0.4	1.77	1.03, 3.04	0.040
UA	0.33	0.12, 0.89	0.028	0.32	0.12, 0.89	0.029
**RAS %DS**						
<50%	—	—				
≥50%	1.41	0.81, 2.46	0.2			
**Echo EF**						
≤40	—	—		—	—	
(40,50]	0.49	0.31, 0.78	0.002	0.46	0.28, 0.76	0.002
(50,60]	0.44	0.27, 0.73	0.002	0.55	0.31, 0.95	0.033
>60	0.27	0.16, 0.46	<0.001	0.38	0.21, 0.71	0.002
**hsCRP**						
≤0.1	—	—				
(0.1,0.2]	1.08	0.63, 1.85	0.8			
(0.2,0.5]	1.12	0.68, 1.83	0.7			
(0.5,82]	1.55	0.95, 2.53	0.080			
**LDL chol**						

	Univariable COX regression			Multivariable COX regression		
**Characteristic**	**HR[1]**	**95% CI[1]**	**p-value**	**HR[1]**	**95% CI[1]**	**p-value**
≤100	—	—				
(100,129]	0.55	0.34, 0.88	0.014			
(129,159]	0.72	0.45, 1.15	0.2			
(159,465]	0.83	0.49, 1.40	0.5			
**Glucose**						
≤80	—	—				
(80,100]	0.95	0.34, 2.64	>0.9			
(100,140]	1.27	0.46, 3.50	0.6			
(140,200]	1.32	0.45, 3.89	0.6			
>200	2.74	0.89, 8.41	0.078			
**eGFR**						
≤60	—	—				
>60	0.29	0.21, 0.42	<0.001			
**suPARnostic [ng/ml]**						
T1	—	—		—	—	
T2	2.68	1.66, 4.33	<0.001	1.68	0.99, 4.33	0.073
T3	3.24	2.03, 5.17	<0.001	3.45	2.11, 5.92	<0.001
**Neutrophile Elastase [ng/ml]**						
< 100 [ng/ml]	—	—				
100-300 [ng/ml]	1.20	0.38, 3.81	0.8			
> 300 [ng/ml]	1.05	0.33, 3.34	>0.9			
**Myeloperoxidase [ng/ml]**						
< 50 [ng/ml]	—	—				
50-100 [ng/ml]	0.97	0.54, 1.72	>0.9			
100-300 [ng/ml]	0.83	0.49, 1.41	0.5			

	Univariable COX regression			Multivariable COX regression		
**Characteristic**	**HR[1]**	**95% CI[1]**	**p-value**	**HR[1]**	**95% CI[1]**	**p-value**
> 300 [ng/ml]	0.88	0.50, 1.55	0.7			
**DNASE1 [ng/ml]**						
< 0.5 [ng/ml]	—	—				
0.5-1 [ng/ml]	0.81	0.55, 1.21	0.3			
> 1 [ng/ml]	0.85	0.56, 1.29	0.4			

[1]HR = Hazard Ratio, CI = Confidence Interval
